# The effects of essential oil from Lippia origanoides and herbal betaine on performance, intestinal integrity, bone mineralization and meat quality in broiler chickens subjected to cyclic heat stress

**DOI:** 10.3389/fphys.2023.1184636

**Published:** 2023-05-30

**Authors:** Roberto Señas-Cuesta, Andressa Stein, Juan D. Latorre, Clay J. Maynard, Xochitl Hernandez-Velasco, Victor Petrone-Garcia, Elizabeth S. Greene, Makenly Coles, Latasha Gray, Lauren Laverty, Kristen Martin, Ileana Loeza, Alvaro J. Uribe, Blanca C. Martínez, Jaime A. Angel-Isaza, Danielle Graham, Casey M. Owens, Billy M. Hargis, Guillermo Tellez-Isaias

**Affiliations:** ^1^ Department of Poultry Science, University of Arkansas, Fayetteville, AR, United States; ^2^ Departamento de Medicina y Zootecnia de Aves, Facultad de Medicina Veterinaria y Zootecnia, UNAM, Ciudad de México, Mexico; ^3^ Departamento de Ciencias Pecuarias, Facultad de Estudios Superiores Cuautitlán UNAM, Cuautitlán, Mexico; ^4^ Promitec, Bucaramanga, Santander, Colombia

**Keywords:** betaine, chickens, essential oils, heat stress, performance

## Abstract

Essential oils (EO) affect performance, intestinal integrity, bone mineralization, and meat quality in broiler chickens subjected to cyclic heat stress (HS). Day-of-hatch Cobb 500 male broiler chicks (*n* = 475) were randomly divided into four groups. Group 1: No heat stress (Thermoneutral) + control diets with no antibiotics; Group 2: heat stress control + control diets; Group 3: heat stress + control diets supplemented with thymol chemotype (45 ppm) and herbal betaine (150 ppm) formulation EO1; Group 4: heat stress + control diets supplemented with phellandrene (45 ppm) and herbal betaine (150 ppm) formulation EO2. From day 10–42, the heat stress groups were exposed to cyclic HS at 35°C for 12 h (8:00–20:00). BW, BWG, FI, and FCRc were measured at d 0, 10, 28, and 42. Chickens were orally gavaged with FITC-d on days 10 (before heat stress) and 42. Morphometric analysis of duodenum and ileum samples and bone mineralization of tibias were done. Meat quality was assessed on day 43 with ten chickens per pen per treatment. Heat stress reduced BW by day 28 (*p* < 0.05) compared to thermoneutral chickens. At the end of the trial, chickens that received both formulations of EO1 and EO2 had significantly higher BW than HS control chickens. A similar trend was observed for BWG. FCRc was impaired by EO2 supplementation. There was a significant increase in total mortality in EO2 compared with EO1 EO1 chickens had lower FITC-d concentrations at day 42 than the HS control. In addition, EO1 treatment is not statistically different if compared to EO2 and thermoneutral. Control HS broilers had significantly lower tibia breaking strength and total ash at day 42 than heat-stressed chickens supplemented with EO1 and EO2. Heat stress affected intestinal morphology more than thermoneutral chickens. EO1 and EO2 improved intestinal morphology in heat-stressed chickens. Woody breast and white striping were more common in thermoneutral chickens than heat stress chickens. In conclusion, the EO-containing diet could improve broiler chicken growth during cyclic heat stress, becoming increasingly relevant in antibiotic-free production in harsh climates.

## 1 Introduction

Heat stress (HS) in chickens is a condition that occurs when chickens are exposed to high temperatures and humidity and are unable to dissipate their body heat efficiently (Khan et al., 2021; Khan et al., 2021). Chickens are homeothermic animals, meaning they have a constant body temperature, which is essential for their normal physiological functions (Abioja and Abiona, 2021). However, when the ambient temperature exceeds their comfort range, chickens can become stressed, leading to a range of negative effects (Uyanga et al., 2023; te Pas et al., 2023; Saracila et al., 2023). During heat stress, chickens can experience a range of symptoms including panting, increased water consumption, reduced feed intake, reduced egg production, decreased growth rate, and increased mortality. In severe cases, heat stress can lead to heat stroke, which can be fatal (Nyoni et al., 2019; Balakrishnan et al., 2023).

There are several ways to mitigate the negative effects of heat stress in poultry, including ventilation and cooling schemes, ensuring access to cool water, adjusting feeding schedules to cooler parts of the day, providing shade in outdoor areas and to avoid handling or transporting chickens during the hottest parts of the day (Brugaletta et al., 2023; Rebez et al., 2023).

Essential oils (EO) may reduce chicken heat stress (Pandey et al., 2019; Yilmaz and Gul, 2023a) due to their antibacterial, anti-inflammatory, and antioxidant effects (Basiouni et al., 2023; Mnisi et al., 2023; Rafeeq et al., 2023). Some of the most used essential oils in poultry include oregano, thyme, rosemary, eucalyptus, cumin, mint, lemon, and cinnamon (Yilmaz and Gul, 2023b; AL-Ramamneh, 2023; Shanthi and Diwan, 2023). The active components of these oils include phenols, terpenes, and flavonoids, which are believed to play a role in their beneficial effects on chicken health (Shehata et al., 2022a).

Several investigators have studied the effects of EO on chickens under heat stress conditions (Shehata et al., 2022b; Yilmaz and Gul, 2023a; Mangan and Siwek, 2023). These studies have shown that EO can improve performance, while reducing the negative outcomes of heat stress, such as reduced water intake, oxidative stress, and inflammation (Chowdhury et al., 2023; Das et al., 2023; Rafeeq et al., 2023). Some EO have improved the immune system of chickens, which can help reduce the incidence of disease under heat stress conditions (Perini et al., 2020; Ruff et al., 2021; Yilmaz and Gul, 2023b; Jahja et al., 2023). Furthermore, certain EO can modulate the expression of genes related to stress response and immune function, which may contribute to their beneficial effects on chicken health (Abo Ghanima et al., 2023; Ding et al., 2023; Wu et al., 2023). Hence, the purpose of the present study was to confirm and extend previous research conducted in our laboratory with the EO of *Lippia origanoides* and betaine from an herbal extract on performance, intestinal integrity, bone mineralization, and meat quality in broiler chickens subjected to cyclic heat stress.

## 2 Materials and methods

### 2.1 Essential oils and herbal betaine

Promitec Santander S.A. (Bucaramanga, Santander, Colombia) provided two formulations of EO with herbal betaine and feed inclusion was based on the manufacturer’s recommendations and analysis. The formulations were:

Formula 1 (EO1): Essential oil of *Lippia origanoides*, thymol chemotype (45 ppm), and herbal betaine (150 ppm). Administration dose: 350 g/Ton food.

Formula 2 (EO2): Essential oil of *Lippia origanoides*, phellandrene chemotype (45 ppm), and herbal betaine (150 ppm). Administration dose: 350 g/Ton food.

The qualitative and quantitative chemical composition of *Lippia origanoides* essential oils for both formulations are summarized in Table 1. Samples of both products were submitted to chromatographic analysis 7890 A (Laboratory of chromatography and mass spectrometry Industrial University of Santander, Bucaramanga, Colombia). The two main components of Formula 1 were thymol (47.5%) and carvacrol (29.9%). On the other hand, the two main components present in Formula 2 were trans-β-Caryophyllene (12.6%) followed by *p*-Cymenene (10.1%). EO’s quick absorption and processing by enterocytes suggests encapsulating feed additives to increase efficiency (Gheisar et al., 2015). In the present study, both formulations were spray-dried maltodextrin microencapsulated to improve encapsulation efficiency, bioavailability, and the lifespan of the EO. Table 2 shows the herbal components of the betaine used in both formulations.

**TABLE 1:** Qualitative and quantitative chemical composition of essential oil of *Lippia origanoides* thymol chemotype, formula 1 (EO1) and essential oil of *Lippia origanoides*, phellandrene chemotype, formula 2 (EO2) by chromatographic analysis.

Compounds EO1 (thymol chemotype)	(%)	Compounds EO2 (phellandrene chemotype)	(%)
Thymol	47.5	trans-β-Caryophyllene	12.6
Carvacrol	29.9	*p*-Cymenene	10.1
ɣ-Terpinen	10.5	α-Humulene	8.1
*p*-Cymenene	10.3	α-Phellandrene	7.6

The LEO (Natbio EsencialPremix^®^) sample was submitted to chromatographic analysis 7890 A (Laboratory of chromatography and mass spectrometry Industrial University of Santander, Bucaramanga, Colombia). Formula 1 (EO1): Essential oil of *Lippia origanoides*, thymol chemotype (45 ppm), and herbal betaine (150 ppm). Administration dose: 350 g/Ton food. Formula 2 (EO2): Essential oil of Lippia origanoides, phellandrene chemotype (45 ppm), and herbal betaine (150 ppm). Administration dose: 350 g/Ton food.

**TABLE 2:** Herbal components of the betaine used in Formula 1 and Formula 2.

Each 100 g of powder contains	Quantity (grams)
*Trigonella foenum-graecum*	25
*Foeniculum vulgare*	25
*Zingiberis officinalis*	20
*Emblica officinalis*	15
*Taraxacum officinalis*	15

Formula 1 (EO1): Essential oil of *Lippia origanoides*, thymol chemotype (45 ppm), and herbal betaine (150 ppm). Administration dose: 350 g/Ton food. Formula 2 (EO2): Essential oil of Lippia origanoides, phellandrene chemotype (45 ppm), and herbal betaine (150 ppm). Administration dose: 350 g/Ton food.

The EO1 and EO2 were included in all three diets and administered since day 1. Starter, grower, and finisher mash diets were used in this experiment and were formulated to approximate the nutritional requirements of broiler chickens as recommended by the National Research Council 1994 and adjusted to the breeder’s recommendations (Cobb-Vantress Inc, 2018). No antibiotics, coccidiostats or enzymes were added to the feed (Table 3).

**TABLE 3:** Ingredient composition and nutrient content of the corn-soybean diets used on as-is basis.

Item	Starter control diet	Grower control diet	Finisher control diet
Ingredients (%)			
Corn	51.85	57.85	59.68
Soybean meal	37.66	31.62	27.23
DDGS 8.1% EE	4.00	4.00	6.00
Poultry fat	3.24	3.44	4.38
Limestone	1.08	1.06	1.03
Dicalcium phosphate	1.01	0.88	0.64
Salt	0.35	0.35	0.31
DL-methionine	0.29	0.25	0.22
L-lysine HCl	0.12	0.13	0.12
Mineral premix^a^	0.10	0.10	0.10
Vitamin premix^b^	0.10	0.10	0.10
L-threonine	0.08	0.09	0.09
Choline chloride	0.06	0.06	0.05
Sodium bicarbonate	0.04	0.05	0.03
Antioxidant^c^	0.02	0.02	0.02
Calculated analysis			
ME (kcal/kg)	3,015.00	3,090.00	3,175.00
Ether extract (%)	5.88	6.20	7.28
Crude protein (%)	22.30	20.00	18.70
Lysine (%)	1.18	1.05	0.95
Methionine (%)	0.59	0.53	0.48
Threonine (%)	0.77	0.69	0.65
Tryptophan (%)	0.25	0.22	0.20
Total calcium (%)	0.90	0.84	0.76
Total phosphorous (%)	0.63	0.58	0.53
Available phosphorus (%)	0.45	0.42	0.38
Sodium (%)	0.20	0.20	0.18
Potassium (%)	1.06	0.94	0.87
Chloride (%)	0.27	0.28	0.25
Magnesium (%)	0.19	0.18	0.17
Copper (%)	19.20	18.46	18.85
Selenium (%)	0.28	0.27	0.26
Linoleic acid (%)	1.01	1.13	1.16

^a^ Mineral premix supplied the following per kg: manganese, 120 g; zinc, 100g; iron, 120 g; copper, 10–15 g; iodine, 0.7 g; selenium, 0.4 g; and cobalt, 0.2 g (Nutra Blend LLC, Neosho, MO, 64850).

^b^ Vitamin premix supplied the following per kg: vitamin A, 20,000,000 IU; vitamin D3, 6,000,000 IU; vitamin E, 75,000 IU; vitamin K3, 9 g; thiamine, 3 g; riboflavin, 8 g; pantothenic acid, 18 g; niacin, 60 g; pyridoxine, 5 g; folic acid, 2 g; biotin, 0.2 g; cyanocobalamin, 16 mg; and ascorbic acid, 200 g (Nutra Blend LLC, Neosho, MO, 64850).

^c^ Ethoxyquin.

### 2.2 Experimental design

Day-of-hatch Cobb 500 male broiler chicks (*n* = 475) were purchased from a commercial hatchery. After arriving, all chicks were vaccinated with a coccidia vaccine (Coccivac®-B52, Merck Animal Health, De Soto, KS 66018), neck tagged, and randomly divided into four groups. Group 1: No heat stress (Thermoneutral-TN) + control diet for starter, grower, and finisher with no antibiotics; Group 2: heat stress control + control diets; Group 3: heat stress + control diets supplemented with EO1 formulation; Group 4: heat stress + control diets supplemented with EO2 formulation. This investigation employed breeder-recommended starter, grower, and finisher diets (Table 3). Diets had no growth boosters. Environmental rooms housed groups. Each room had two 150 × 300 cm pens with feeding and watering systems. Five replicates per treatment were under heat stress circumstances, and four replicates were under thermoneutral (TN) conditions with 25 birds/pen. TN chicks were raised in commercial production conditions (temperature, light). From days 15–42, the temperature was gradually reduced from 32°C to 24°C with relative humidity at 55% ± 5%. From day 10–42, the heat stress groups were exposed to cyclic heat stress at 35°C for 12 h (from 8:00 to 20:00 h). On day 18, eight chickens were randomly selected to orally insert a Thermochron temperature logger (iButton, DS 1922L, Embedded Data Systems, Lawrenceburg, KY). The devices measured body temperature in the gizzard, according to Flees et al. (2017). Chickens' body temperatures were recorded every minute for the first 2 hours and every hour after heat stress. BW, BWG, FI, and FCRc were measured at days 0, 10, 28, and 42. Ten chickens each pen (*n* = 40 TN; *n* = 50 heat stress) per group were processed on day 43 to evaluate processing characteristics and meat quality. Protocol 16,084 of the University of Arkansas Institutional Animal Care and Use Committee supervised animal care.

Four random chickens per pen (*n* = 16 TN; *n* = 20 heat stress) were orally gavaged with 8.32 mg/kg BW of fluorescein isothiocyanate-dextran (FITC-d, MW 3–5 kDa; Sigma-Aldrich Co.) on day 10 (before heat stress) and day 42 to assess intestinal permeability as stated by Baxter et al. (2017). On the same days, both tibias were collected to evaluate break strength (kg) and total ash (%) as described by Gautier et al. (2017).

On the evaluation day, birds were euthanized, and ileum and duodenum samples were taken (*n* = 8) for enteric morphometric analysis. Each bird’s middle duodenum and lower ileum were excised and fixed in 10% buffered formaldehyde for 48 h. A 5-μm section of each intestine segment was embedded in paraffin, mounted on a glass slide, and stained with hematoxylin and eosin for light microscope analysis. All morphological parameters were measured using ImageJ (http://rsb.info.nih.gov/ij/). The average results from each sample’s 10 replicate measurements for each variable were used in statistical analysis as described by Aptekmann et al., 2001 and Sakamoto et al., 2000.

Broilers were reared under chronic heat stress conditions for 42 days. At day of processing (day 43), 10 birds per pen were selected., after a 10-h feed withdrawal period and processed as described by Mehaffey et al. (2006). After a 0.25 h prechill at 12°C, carcasses were immersed in 0°C tanks for 2.5 h with manual agitation. Carcasses were deboned at 3 h postmortem to weigh the breast, tender, wing, and complete leg. Myopathy score (woody breast and white striping), WHC, color, and pH were used to analyze pectoralis major muscles following the procedures described by Kuttappan et al. (2012); Tijare et al. (2016), respectively. After measuring and scoring myopathies, fillets were placed on white plastic storage trays, wrapped in plastic overlay liners, and kept in a walk-in refrigerator at 4°C until 24 h postmortem. Breast fillets were weighed 24 h postmortem to determine drip loss. Drip loss was estimated as a weight-to-deboned weight proportion.

### 2.3 Data and statistical analysis

Data were analyzed using the JMP Pro 16.0 platform (SAS Institute, United States) according to a Randomized Complete Block Design (RCBD). All data were subjected to analysis of variance (ANOVA). Each pen was considered as the experimental unit for performance parameters. Individual birds were considered as the experimental unit for histological measurements, serum FITC-d analysis, bone quality parameters, and meat quality characteristics. Statistical significance was set at *p* ≤ 0.05. If significance was met, means were separated using a Tukey’s HSD test.

## 3 Results

The results of the evaluation of EO and herbal betaine on performance parameters for broiler chickens exposed to cyclic heat stress are summarized in Table 4. By day 28 (18 days after initiating heat stress, groups subjected to heat stress showed a significant (*p* < 0.05) reduction in BW compared with thermoneutral chickens. However, at the end of the trial (day 42), chickens that received both formulations EO1 and EO2, and were exposed to cyclic heat stress, showed a significant improvement (*p* < 0.05) in BW compared to heat stress control chickens. A similar trend was observed for BWG. FCRc was impaired by EO2 supplementation and FI increased only in EO2, not in EO1. There was a significant increase in total mortality in EO2 compared with EO1 (Table 4).

**TABLE 4:** Evaluation of essential oils (EO) and herbal betaine on performance parameters of broiler chickens exposed to cyclic heat stress^a^.

Performance	Thermoneutral	Heat stress	Heat stress + EO1	Heat stress + EO2
Body weight (g)				
0	41.1 ± 0.39	41.2 ± 0.23	40.8 ± 0.29	41.3 ± 0.33
10	262.9 ± 8.39^ab^	268.4 ± 4.97^a^	242.2 ± 10.81^b^	253.8 ± 4.92^ab^
28	1,607.8 ± 23.76^a^	1,393.2 ± 17.78^b^	1,325.4 ± 51.49^b^	1,365.5 ± 28.11^b^
42	3,187.2 ± 7.89^a^	2,482.5 ± 84.42^c^	2,650.3 ± 37.14^b^	2,639.3 ± 25.70^b^
Body weight gain (g)				
0–10	221.0 ± 8.72^ab^	226.4 ± 4.52^a^	200.4 ± 10.51^b^	211.8 ± 4.84^ab^
0–28	1,553.3 ± 23.84^a^	1,342.0 ± 17.10^b^	1,274.4 ± 50.91^b^	1,314.4 ± 28.94^b^
0–42	3,130.3 ± 8.52^a^	2,428.4 ± 83.43^c^	2,592.0 ± 37.81^b^	2,579.2 ± 23.01 ^bc^
Feed intake (g)				
0–10	265.8 ± 5.74^b^	270.0 ± 3.27^b^	265.7 ± 17.10^b^	314.6 ± 15.80^a^
0–28	2,330.1 ± 52.77^a^	1987.3 ± 35.47 ^bc^	1936.8 ± 61.41^c^	2,118.3 ± 62.38^b^
0–42	5,247.8 ± 43.60^a^	4,275.0 ± 181.64^b^	4,490.4 ± 108.15^b^	5,150.4 ± 166.47^a^
FCRc				
0–10	1.007 ± 0.039^b^	1.002 ± 0.01^b^	1.096 ± 0.06 ^ab^	1.235 ± 0.07^a^
0–28	1.435 ± 0.027	1.414 ± 0.03	1.459 ± 0.03	1.548 ± 0.07
0–42	1.626 ± 0.013^b^	1.692 ± 0.01^b^	1.691 ± 0.03^b^	1.844 ± 0.06^a^
Total Mortality (%)	2.0^ab^	2.4^ab^	1.6^b^	7.2^a^

^a^ Data are expressed as the mean ± SE.

abc Indicates significant differences between the treatments within the rows (*p* < 0.05). Cyclic heat stress started at day 10. EO1: essential oil of *Lippia origanoides*, thymol chemotype (45 ppm) and herbal betaine (150 ppm). Administration dose: 350 g/Ton food. EO2: essential oil of *Lippia origanoides*, phellandrene chemotype (45 ppm) and herbal betaine (150 ppm). Administration dose: 350 g/Ton food.

Two hours after introducing heat stress into the experimental groups, chickens' body core temperatures increased significantly (*p* < 0.05) and remained raised during heat stress throughout the trial (data not shown).

Table 5 expresses the evaluation of EO and herbal betaine on broiler chickens exposed to cyclic heat stress on serum FITC-d and bone parameters at days 10 and 42. No significant differences were observed for serum FITC-d or bone parameters at day 10. However, at day 42, a significant difference (*p* < 0.05) in the concentration of FITC-d was observed in chickens that received EO1 compared to HS control. No significant difference between EO1 and EO2 to as concern FITC-d levels. Control HS chickens showed a significant reduction in tibia break strength and total tibia ash (%) at day 42 compared with chickens exposed to heat stress while being supplemented with EO1 and EO2 (Table 5).

**TABLE 5:** Evaluation of essential oils (EO) and herbal betaine on broiler chickens exposed to cyclic heat stress on serum Fluorescein isothiocyanate–dextran (FITC-d) and bone parameters at days10 and 42^a^.

	Thermoneutral	Heat stress	Heat stress + EO1	Heat stress + EO2
Serum FITC-d (ng/mL)				
d 10	39.2 ± 6.42	36.9 ± 10.07	33.6 ± 7.89	37.1 ± 7.71
d 42	50.9 ± 13.46^c^	114.5 ± 13.45^a^	67.4 ± 12.46^bc^	108.8 ± 21.98^ab^
Tibia break strength (kg)				
d 10	17.3 ± 0.45	16.5 ± 0.32	16.8 ± 0.30	17.1 ± 0.33
d 42	33.2 ± 0.53^a^	22.3 ± 0.63^c^	30.2 ± 0.70^b^	30.9 ± 0.58^b^
Total ash from tibia (%)				
d 10	53.9 ± 0.65	52.7 ± 0.73	53.2 ± 0.81	53.5 ± 0.71
d 42	56.0 ± 0.90^a^	52.2 ± 0.83^d^	54.2 ± 0.93^b^	53.1 ± 0.76^c^

^a^ Data are expressed as the mean ± SE.

abc Indicates significant differences between the treatments within the rows (*p* < 0.05). Cyclic heat stress started at day 10. EO1: essential oil of *Lippia origanoides*, thymol chemotype (45 ppm) and herbal betaine (150 ppm). Administration dose: 350 g/Ton food. EO2: essential oil of *Lippia origanoides*, phellandrene chemotype (45 ppm) and herbal betaine (150 ppm). Administration dose: 350 g/Ton food.

The results of the evaluation of EO and herbal betaine on morphometric analysis of the duodenum and ileum mucosa of broiler chickens exposed to cyclic heat stress at day 42 are summarized in Table 6. In the duodenum, heat stress had a severe effect (*p* < 0.05) on villus height, crypt depth, villus width, villus-to-crypt ratio and villus surface area index when compared to thermoneutral chickens. Nevertheless, the severity of the heat stress was reduced (*p* < 0.05) in chickens that were supplemented with EO1 and EO2, and a similar trend was observed in the ileum (Table 6).

**TABLE 6:** Evaluation of essential oils (EO) and herbal betaine on morphometric analysis of the duodenum and ileum mucosa of broiler chickens exposed to cyclic heat stress at day 42^a^.

Treatments	Villus height (µm)	Crypt depth (µm)	Villus width (µm)	Villus: crypt ratio	Villus surface area index (mm^2^)
Duodenum					
Thermoneutral	976.7 ± 17.0^a^	187.5 ± 70.4^a^	179.8 ± 12.5^a^	5.9 ± 0.8^a^	538.4 ± 20.1^a^
Heat Stress	706.8 ± 40.6^c^	145.0 ± 70.7^c^	140.1 ± 24.5^c^	4.1 ± 0.6^c^	381.8 ± 21.5^c^
Heat Stress + EO1	853.8 ± 53.7^b^	160.5 ± 32.7^b^	158.2 ± 90.3^b^	5.2 ± 0.5^b^	405.6 ± 23.0^b^
Heat Stress + EO2	838.7 ± 83.9^b^	160.1 ± 37.8^b^	161.8 ± 19.2^b^	5.4 ± 0.9^b^	438.5 ± 24.5^b^
Ileum					
Thermoneutral	551.3 ± 52.4^a^	118.6 ± 40.5^a^	28.5 ± 81.7	5.9 ± 0.5^a^	18.4 ± 7.2^a^
Heat Stress	324.1 ± 94.1^c^	206.7 ± 52.4^c^	27.0 ± 81.0	1.5 ± 0.5^c^	15.7 ± 8.1^b^
Heat Stress + EO1	431.9 ± 93.2^b^	157.4 ± 33.5^b^	29.1 ± 61.3	2.7 ± 0.5^b^	16.8 ± 7.5^b^
Heat Stress + EO2	425.2 ± 94.2^b^	149.4 ± 38.6^b^	33.2 ± 30.4	2.8 ± 0.5^b^	18.9 ± 9.0^b^

^a^ Values were expressed as means ± SE, representing 8 birds/group and 10 measurements/parameter/bird.

^abc^ Indicates significant differences between the treatments within the columns (*p* < 0.05). Cyclic heat stress started at day 10. EO1: essential oil of *Lippia origanoides*, thymol chemotype (45 ppm) and herbal betaine (150 ppm). Administration dose: 350 g/Ton food. EO2: essential oil of *Lippia origanoides*, phellandrene chemotype (45 ppm) and herbal betaine (150 ppm). Administration dose: 350 g/Ton food.

Table 7 expresses the results of the evaluation of EO and herbal betaine on final body weight and carcass yields of broiler chickens exposed to cyclic heat stress at day 43. At processing, it was clear that heat stress had a negative impact (*p* < 0.05) on the live weight of the chickens compared to thermoneutral chickens. However, chickens that were supplemented with EO1 or EO2, showed a significant improvement (*p* < 0.05) in BW compared with HS control chickens. No differences were observed (*p* > 0.05) in fat yield (%), hot carcass (%), or cold carcass (%) between treatments (Table 7). Thermoneutral chickens and chickens supplemented with EO1, showed a significant reduction (*p* < 0.05) in wing yield. Nevertheless, breast yield in thermoneutral chickens was significantly higher *(p* < 0.05) than in chickens exposed to heat stress. Leg quarter yield was reduced (*p* < 0.05) in thermoneutral chickens compared to heat stress chickens. No differences (*p* > 0.05) were observed in tender yield or rack yield between treatments (Table 7).

**TABLE 7:** Evaluation of essential oils (EO) and herbal betaine on final body weight (g) and carcass yields (%) of broiler chickens exposed to cyclic heat stress at day 43^a^.

	Thermoneutral	Heat stress	Heat stress + EO1	Heat stress + EO2
Live weight	3,310.73 ± 30.02^a^	2,468.60 ± 26.85^c^	2,718.54 ± 26.85^b^	2,673.10 ± 26.85^b^
Fat yield (%)	1.11 ± 0.05	1.05 ± 0.04	1.04 ± 0.04	1.01 ± 0.04
Hot carcass yield (%)	74.73 ± 0.21	74.66 ± 0.18	74.26 ± 0.18	74.22 ± 0.18
Chilled carcass yield (%)	79.37 ± 0.36	79.06 ± 0.32	78.81 ± 0.32	79.22 ± 0.32
Wing yield (%)	8.08 ± 0.08^b^	8.70 ± 0.07^a^	8.30 ± 0.07^b^	8.61 ± 0.07^a^
Breast yield (%)	20.46 ± 0.20^a^	17.50 ± 0.18^b^	18.09 ± 0.18^b^	18.10 ± 0.18^b^
Tender yield (%)	4.03 ± 0.07	4.03 ± 0.06	4.00 ± 0.06	4.14 ± 0.06
Leg yield (%)	23.96 ± 0.19^b^	25.75 ± 0.17^a^	25.32 ± 0.17^a^	25.36 ± 0.17^a^
Rack yield (%)	21.67 ± 0.18	22.03 ± 0.16	21.84 ± 0.16	21.72 ± 0.16

^a^ Data are expressed as the mean ± SE.

^abc^ Indicates significant differences between the treatments within the rows (*p* < 0.05). Cyclic heat stress started at day 10. EO1: essential oil of *Lippia origanoides*, thymol chemotype (45 ppm) and herbal betaine (150 ppm). Administration dose: 350 g/Ton food. EO2: essential oil of *Lippia origanoides*, phellandrene chemotype (45 ppm) and herbal betaine (150 ppm). Administration dose: 350 g/Ton food.

The results of the evaluation of EO and herbal betaine on broiler breast myopathy scores and breast myopathy percent incidence of broiler chickens exposed to cyclic heat stress at day 43 are summarized in Table 8. As expected, thermoneutral chickens showed significantly higher incidences (*p* < 0.05) of both myopathies, when compared to heat stress chickens, regardless of the dietary inclusion of EO1 or EO2 (Table 8).

**TABLE 8:** Evaluation of essential oils (EO) and herbal betaine on broiler breast myopathy scores and breast myopathy percent incidence of broiler chickens exposed to cyclic heat stress at day 43^a^.

	Thermoneutral	Heat stress	Heat stress + EO1	Heat stress + EO2
Woody Breast^1^				
Average	0.74 ± 0.04^a^	0.21 ± 0.04^b^	0.32 ± 0.04^b^	0.27 ± 0.04^b^
0 Occurrence	47.50 ± 5.09^b^	98.00 ± 4.55^a^	94.00 ± 4.55^a^	98.00 ± 4.55^a^
1 Occurrence	52.50 ± 5.09^a^	2.00 ± 4.55^b^	6.00 ± 4.55^b^	2.00 ± 4.55^b^
2 Occurrence	—	—	—	—
White Striping2				
Average	1.18 ± 0.05^a^	0.96 ± 0.04^b^	1.03 ± 0.04 ^ab^	0.95 ± 0.04^b^
0 Occurrence	5.00 ± 4.12	16.00 ± 3.69	8.00 ± 3.69	14.00 ± 3.69
1 Occurrence	80.00 ± 5.03	82.00 ± 4.50	86.00 ± 4.50	84.00 ± 4.50
2 Occurrence	15.00 ± 3.11^a^	2.00 ± 2.78^b^	6.00 ± 2.78 ^ab^	2.00 ± 2.78^b^

^a^ Data are expressed as the mean ± SE., ab Indicates significant differences between the treatments within the rows (*p* < 0.05). Cyclic heat stress started at day 10. EO1: essential oil of Lippia origanoides, thymol chemotype (45 ppm) and herbal betaine (150 ppm). Administration dose: 350 g/Ton food. EO2: essential oil of Lippia origanoides, phellandrene chemotype (45 ppm) and herbal betaine (150 ppm). Administration dose: 350 g/Ton food. For average myopathy scores, thermoneutral treatments only consisted of an n = 40. All remainders had an n = 50. For percent incidence, scores were broken out on a pen basis and consisted of an n = 24. 1Breast fillets were considered a score of 0, 1, or 2 for woody breast if the fillet was flexible throughout, stiff in cranial region, or stiff in the cranial and caudal regions, respectively. 2Breast fillets were considered a score of 0, 1, or 2 for white striping if the fillet displayed no visible stripes, stripes less than 1 mm, or stripes larger than 1 mm, respectively.

Table 9 expresses the results of the evaluation of EO and herbal betaine on raw meat quality parameters of broiler chickens exposed to cyclic heat stress at day 43. Control HS chickens showed a significant reduction (*p* < 0.05) in drip loss (%) compared with thermoneutral and EO2 supplemented chickens. However, chickens supplemented with EO2 expressed a significant reduction (*p* < 0.05) in the color measurement of relative lightness (L*) compared to thermoneutral chickens. In contrast, color measurements for relative yellowness (b*) were significantly higher (*p* < 0.05) in thermoneutral chickens (Table 9).

**TABLE 9:** Evaluation of essential oils (EO) and herbal betaine on raw meat quality parameters of broiler chickens exposed to cyclic heat stress at day 43^a^.

	Thermoneutral	Heat stress	Heat stress + EO1	Heat stress + EO2
Drip loss (%)^b^	1.24 ± 0.13^a^	0.75 ± 0.11^b^	0.98 ± 0.12^ab^	1.20 ± 0.11^a^
pH	5.77 ± 0.03	5.76 ± 0.03	5.78 ± 0.03	5.72 ± 0.03
L^a^ ^c^	60.96 ± 0.47^a^	59.70 ± 0.42 ^ab^	59.56 ± 0.42 ^ab^	58.95 ± 0.42^b^
a^a^ ^d^	3.31 ± 0.19	3.25 ± 0.17	3.24 ± 0.17	3.21 ± 0.17
b^a^ ^e^	8.23 ± 0.27^a^	6.99 ± 0.24^b^	7.23 ± 0.24^b^	7.12 ± 0.24^b^

^a^ Data are expressed as the mean ± SE.

ab Indicates significant differences between the treatments within the rows (*p* < 0.05). Cyclic heat stress started at day 10. EO1: essential oil of *Lippia origanoides*, thymol chemotype (45 ppm) and herbal betaine (150 ppm). Administration dose: 350 g/Ton food. EO2: essential oil of *Lippia origanoides*, phellandrene chemotype (45 ppm) and herbal betaine (150 ppm). Administration dose: 350 g/Ton food.

^b^ Drip loss—Measured as percent loss in relation to deboned part weight. Presented as percent loss.

c L*—CIE, color measurement of relative lightness. Measured 0-100 with 0 being black and 100 being white in color.

^d^ a*—CIE, color measurement of relative redness. Measured −60, to +60 with −60, being green and +60 being red in color.

^e^ b*—CIE, color measurement of relative yellowness. Measured −60, to +60 with −60, being blue and +60 being yellow in color.

## 4 Discussion

Colombia is a megadiverse nation, as it is home to more than 45,000 unique plant species. A current program for the development of the agro-industrial sector in Colombia investigates sustainable methods for extracting plant metabolites from native plants such as *Lippia. alba*, and *Lipia origanoides*. EO of various *Lippia* species possess antimalarial, sedative, hypotensive, and anti-inflammatory properties (Pascual et al., 2001; Stashenko et al., 2010). Nevertheless, the EO composition changes during plant development, cultivation conditions, or phenotypic plasticity (Antolinez-Delgado and Rodríguez-López, 2008). Thus, chemotype differentiation requires molecular biology and secondary metabolite analysis. In Colombia, infusions of leaves and flowers of *L. origanoides* are used in popular medicine, to treat digestive disorders (Pascual et al., 2001). Chromatographic analyses of EO from *Lippia origanoides* plants growing in the wild throughout various Colombian regions have identified 139 substances (Stashenko et al., 2010). Differential identification of these EO and extracts classifies *L. origanoides* into three chemotypes based on their essential oil primary components (Stashenko et al., 2010). Chemotype A had phellandrene, p-cymene, and limonene, while B and C had carvacrol and thymol (Curado et al., 2006).

The qualitative and quantitative chemical composition of *Lippia origanoides* essential oils for both formulations submitted to chromatographic analysis 7890 A revealed that the two main components of *L. origanonides* in Formula 1 were thymol (47.5%) and carvacrol (29.9%). On the other hand, the two main components of *L. origanonides* present in Formula 2 were trans-β-Caryophyllene (12.6%) followed by *p*-Cymenene (10.1%), confirming clear differences in the primary components of essential oils in both chemotypes of *L. origanonides*.

The EOs have gained popularity as a potential strategy for mitigating the effects of heat stress in poultry (Raza et al., 2022). In this study, we evaluated the potential effects of two formulations of EO from *L. origanoides* chemotype thymol (EO1) and *L. origanoides* chemotype phellandrene (EO2) combined with herbal betaine on chickens under cyclic heat stress conditions. The EO from *L. origanoides* have shown a wide range of biological activities, including antimicrobial, anti-inflammatory, and antioxidant properties on chickens under heat stress conditions, suggesting that the EO contained in *L. origanoides* can reduce the severity of the negative effects of heat stress in broiler chickens (Turcu et al., 2018).

The mechanisms behind the effects of EOs on the performance of chickens under heat stress are not yet fully understood. However, several potential mechanisms have been proposed. Essential oils can improve gut morphology, increase the activity of digestive enzymes, and enhance the production of beneficial gut microbes. They have also been shown to have antioxidant and anti-inflammatory properties in chickens leading to improved performance (Gholami-Ahangaran et al., 2022).

Betaine (TMG) detoxifies homocysteine and methylates. Many naturally occurring betaines protect cells from osmotic stress as organic osmolytes (Van Puyvelde et al., 2023). Betaine inside cells prevents dehydration (Denaxa et al., 2023). Enzymes, proteins, and membranes are unaffected by this buildup. Betaine is a growing biological methyl donor (Bekdash, 2023). Betaine increases energy metabolism enzyme activity and reduces liver fat in hens (Sun L et al., 2023). Betaine-supplemented hens have improved growth rate, feed efficiency, and carcass quality, especially during heat stress or disease outbreaks (Suliman et al., 2023; Sun S et al., 2023).

EO1 and EO2 from Lippia origanoides were combined with herbal betaine in this investigation. Maltodextrin microencapsulated both formulations to protect and improve EO bioavailability. Heat stress decreased performance compared to thermoneutral chicks in this study. EO1 and EO2 had higher BW than HS chickens at the end of the research. BWG improved significantly in EO1-supplemented chickens. EO2-supplemented birds devoured more feed, lowering FCRc, but BWG was not different.

Essential oils are replacing antibiotics in poultry health and growth (Booth and van Vuuren, 2023). Chicken health and performance depend on intestinal permeability, the gut lining’s ability to absorb nutrients and block harmful substances from entering the bloodstream (Gilani et al., 2021; Rocchi et al., 2022).

Thymol increases chicken intestine tight junction protein expression and localization, improving intestinal barrier function (Roussel et al., 2015; Wei et al., 2017). Thymol may also modulate tight junctions through its anti-inflammatory effects (Yao et al., 2018; Pham et al., 2023) contributing to maintain the integrity of tight junctions in the chicken intestine.

Moreover, several investigators have demonstrated that betaine also improves gut integrity by regulation of tight junctions in the intestinal epithelium of chickens (Shin et al., 2018; Shin et al., 2018; Wu et al., 2020; El-Chami et al., 2021). In this investigation, chickens under heat stress that received EO1 had a substantial reduction in serum FITC-d at day 42, a well-known intestinal permeability biomarker.

Strong bones require complicated bone mineralization. Bone health affects eggshell quality, mobility, and overall health in hens (Talaty et al., 2009). EO from Lippia origanoides improved bone mineralization in chicken tibias exposed to cyclic heat stress (Ruff et al., 2021). By increasing calcium absorption and decreasing bone resorption, thymol may improve bone mineralization. Alagawany et al. (2018) examined how thymol affects laying hen bone quality. For 12 weeks, chickens drank 0.05%, 0.10%, or 0.15% thymol. Thymol enhanced bone microstructure and mineral density. Thymol may improve bone health by increasing osteoblast and decreasing osteoclast activity (Ghanima et al., 2020).

Betaine supplementation increases bone mineral density and bone microstructure, which can prevent fractures and improve skeletal health by making minerals like calcium and phosphorus more available (Kuo et al., 2023). Compared to HS control hens, cyclic heat stress-exposed chicks fed with EO1 and EO2 had significantly higher tibia breaking strength and total ash.

The duodenum is essential for digestion and absorption. Duodenum cells are columnar. The lamina propria contains blood arteries, lymphatic vessels, and immunological cells. Lamina propria smooth muscle cells move villi to receive nutrition (Pelicano et al., 2005). Villi cover enterocytes. These villi increase duodenal nutrition absorption surface area. Villus height depends on villus cell proliferation, differentiation, and tip cell shedding. Infections, inflammation, nutrient deficiencies, and environmental contaminants affect villi height. Villi height decreases with malabsorption and gastrointestinal illnesses (Babbin et al., 2006).

Thymol improves duodenal villi shape and function (Mo et al., 2023). Thymol protects duodenal villi from oxidative stress (He et al., 2017). When free radicals outnumber antioxidants, oxidative stress ensues (Basiouni, et al., 2023). Free radicals destroy cells and tissues, causing inflammation and disease. Antioxidants help to neutralize free radicals and protect cells from damage (Shehata et al., 2022b). Similarly, betaine supplementation has been shown to increase villi height in the duodenum of chickens by increasing the secretion of mucus and improving the integrity of the intestinal mucosal barrier in chickens under long-term heat stress (Liu et al., 2019). The results of the present study confirm the adverse effects of heat stress on the villus height. Nevertheless, chickens that received both formulations of EO showed significantly higher villus in both the duodenum and ileum compared to HS control chickens.

Intestinal crypts, which produce intestinal stem cells, are invaginations between villi. Broiler chickens' intestinal health is determined by their villus height to crypt depth ratio, which indicates the small intestine’s ability to absorb nutrients (Peng et al., 2022). Thymol supplementation improves this histomorphological ratio in broiler chickens (Galli et al., 2020). According to earlier studies, the increased villus-to-crypt depth ratio in the duodenum and ileum of hens given EO1 or EO2 may improve nutritional absorption, development, and production (Peng et al., 2016; Magouz et al., 2022).

Carcass yield is the amount of chicken flesh received after slaughtering and processing. It affects chicken farming profitability and the amount of meat sold. Chickens fed EO or betaine exhibit higher carcass yields (Gumus and Gelen, 2023; Pardo et al., 2023; Zheng et al., 2023). Heat stress reduces carcass yield, increasing leg meat yield at the expense of breast meat yield and meat quality (Zaboli et al., 2019; Greene et al., 2021). In this investigation, EO and betaine did not improve chickens' cyclic heat stress severity. Heat stress lowered feed intake and growth compared to thermoneutral chicks. Thus, thermoneutral chickens had more myopathies. Myopathies also contain a yellow viscous fluid that increases instrumental yellowness (b*; Sihvo et al., 2014; Maynard et al., 2023). In this experiment, thermoneutral broilers had a greater b* value than heat stress broilers regardless of EO treatment.

In summary, the dietary formulation with the tested EOs may be a viable nutritional strategy to support the growth performance of broiler chickens exposed to cyclic heat stress, which is becoming increasingly important in antibiotic-free production carried out in adverse climate. However, the potential benefits of using essential oils in combination with other management strategies for heat-stressed chickens should not be ignored. Further studies to evaluate the effects of these formulations on mitochondria function, gene expression of inflammatory and tight junction proteins, and intestinal and respiratory microbiomes are currently being investigated.

## Data Availability

The original contributions presented in the study are included in the article/supplementary material, further inquiries can be directed to the corresponding author.
